# A genotyping assay to determine geographic origin and transmission potential of *Plasmodium falciparum* malaria cases

**DOI:** 10.1038/s42003-021-02667-0

**Published:** 2021-09-30

**Authors:** Alvaro Molina-Cruz, Nadia Raytselis, Roxanne Withers, Ankit Dwivedi, Peter D. Crompton, Boubacar Traore, Giovanna Carpi, Joana C. Silva, Carolina Barillas-Mury

**Affiliations:** 1 Laboratory of Malaria and Vector Research, National Institute of Allergy and Infectious Diseases, National Institutes of Health, Rockville, MD 20852 USA; 2 Institute for Genome Sciences, University of Maryland School of Medicine, Baltimore, MD 21201 USA; 3 Malaria Infection Biology and Immunity Section, Laboratory of Immunogenetics, National Institute of Allergy and Infectious Diseases, NIH, Rockville, MD 20852 USA; 4 Mali International Center of Excellence in Research, University of Sciences, Techniques and Technologies of Bamako, Bamako, Mali; 5 Department of Biological Sciences, Purdue University, West Lafayette, IN 47907 USA; 6 Department of Microbiology and Immunology, University of Maryland School of Medicine, Baltimore, MD 21201 USA

**Keywords:** Population genetics, Genotyping and haplotyping

## Abstract

As countries work towards malaria elimination, it is important to monitor imported cases to prevent reestablishment of local transmission. The *Plasmodium falciparum Pfs47* gene has strong geographic population structure, because only those parasites with *Pfs47* haplotypes compatible with the mosquito vector species in a given continent are efficiently transmitted. Analysis of 4,971 world-wide *Pfs47* sequences identified two SNPs (at 707 and 725 bp) as sufficient to establish the likely continent of origin of *P. falciparum* isolates. *Pfs47* sequences from Africa, Asia, and the New World presented more that 99% frequency of distinct combinations of the SNPs 707 and 725 genotypes. Interestingly, Papua New Guinea *Pfs47* sequences have the highest diversity in SNPs 707 and 725. Accurate and reproducible High-Resolution Melting (HRM) assays were developed to genotype *Pfs47* SNPs 707 and 725 in laboratory and field samples, to assess the geographic origin and risk of local transmission of imported *P. falciparum* malaria cases.

## Abstract (web summary)

Molina-Cruz et al. analyzed 4,971 sequences of the *Plasmodium falciparum Pfs47* gene and identified a combination of two SNP haplotypes highly correlated with geographic origin and transmission potential by mosquito vectors. A simple High-Resolution Melting RT-PCR assay was developed to genotype these SNPs and establish the likely continent of origin and transmission risk of *P. falciparum* isolates.

## Introduction

Malaria in humans is caused by *Plasmodium* parasites and, as a result of global elimination efforts, the disease burden has decreased considerably in the last 20 years, with a decrease of 29% in the incidence of malaria and of 60% in the mortality rate. However, despite these recent advances, malaria still infects more than 220 million people every year, leading to an estimated 409,000 deaths in 2019^1^, the majority caused by *Plasmodium falciparum* in sub-Saharan Africa. The WHO Global Malaria Program coordinates international efforts to control and eliminate malaria and has already certified several countries as malaria-free^1^. Once local elimination is achieved, preventing the reintroduction of the disease becomes the primary focus of national malaria control programs^2^. Since the local mosquito vectors of malaria are still present after the parasite is eliminated^3^, imported malaria cases have the potential to give rise to new outbreaks, particularly as immunity to the parasite in the population decreases over time^4^. Given extensive and ever-growing international human mobility, constant monitoring for potential resurgence of malaria infections and assessing the risk of local transmission are critical measures to guide a cost-effective response to imported malaria cases. Thus, there is need for practical assays with informative *Plasmodium* genetic markers to predict the transmission risk of an imported malaria case in a given region.

Malaria is transmitted by mosquitoes of the genus *Anopheles*, and different anopheline species tend to be restricted to distinct biogeographic regions or continents^3^. The globalization of *P. falciparum* malaria out of Africa^5^, involved the adaptation of the parasite to more than 70 different anopheline vector species^6^. Several lines of evidence indicate that adaptation of *P. falciparum* to evolutionary distant vectors resulted in strong natural selection on *Pfs47*^7^, a gene mediating the parasite’s ability to evade the mosquito immune system, and therefore critical for the parasite’s survival and transmission^8^. Consistent with natural selection by mosquito vectors, *Pfs47* has a strong geographic population structure, with different haplotypes predominating in continents harboring distinct vector species^7,9,10^. Controlled experimental infections have directly shown that *P. falciparum* from a given continent infects sympatric vectors more efficiently when compared to vectors from a different continent^7^. Furthermore, the compatibility of a *P. falciparum* line with a given mosquito species can be modified by exchanging the *Pfs47* haplotype the parasite carries^7^. We have shown that *Pfs47* interacts with a specific mosquito midgut receptor, and the in vitro binding affinity of different *P. falciparum Pfs47* haplotypes, with the receptor in a given mosquito species, is consistent with the parasite/vector compatibility observed in vivo^7,11^. Therefore, the interaction of *Pfs47* haplotypes with different vector species follows a “lock-and-key” model. This model proposes that those parasites expressing a *Pfs47* haplotype compatible with the *Pfs47* receptor of a given vector can avoid activating an immune response, survive, complete their development in the mosquito, and be transmitted to a new human host^7^.

Here, we analyzed a large number of *Pfs47* sequences collected around the world, to identify combinations of *Pfs47* gene polymorphisms unique to a given continent, which could be used as molecular markers to establish the geographic origin and transmission potential of malaria cases of unknown origin. Simple and low-cost PCR-based high-resolution melting (HRM) assays were developed to facilitate the genotyping of the two most informative *Pfs47* single nucleotide polymorphisms (SNPs). We provide direct evidence that the combination of these two *Pfs47* SNPs can be easily determined with these assays and makes it possible to establish the geographic origin of *P. falciparum* isolates, to assess the risk of local transmission of imported malaria cases.

## Results

**Fig. 1:**
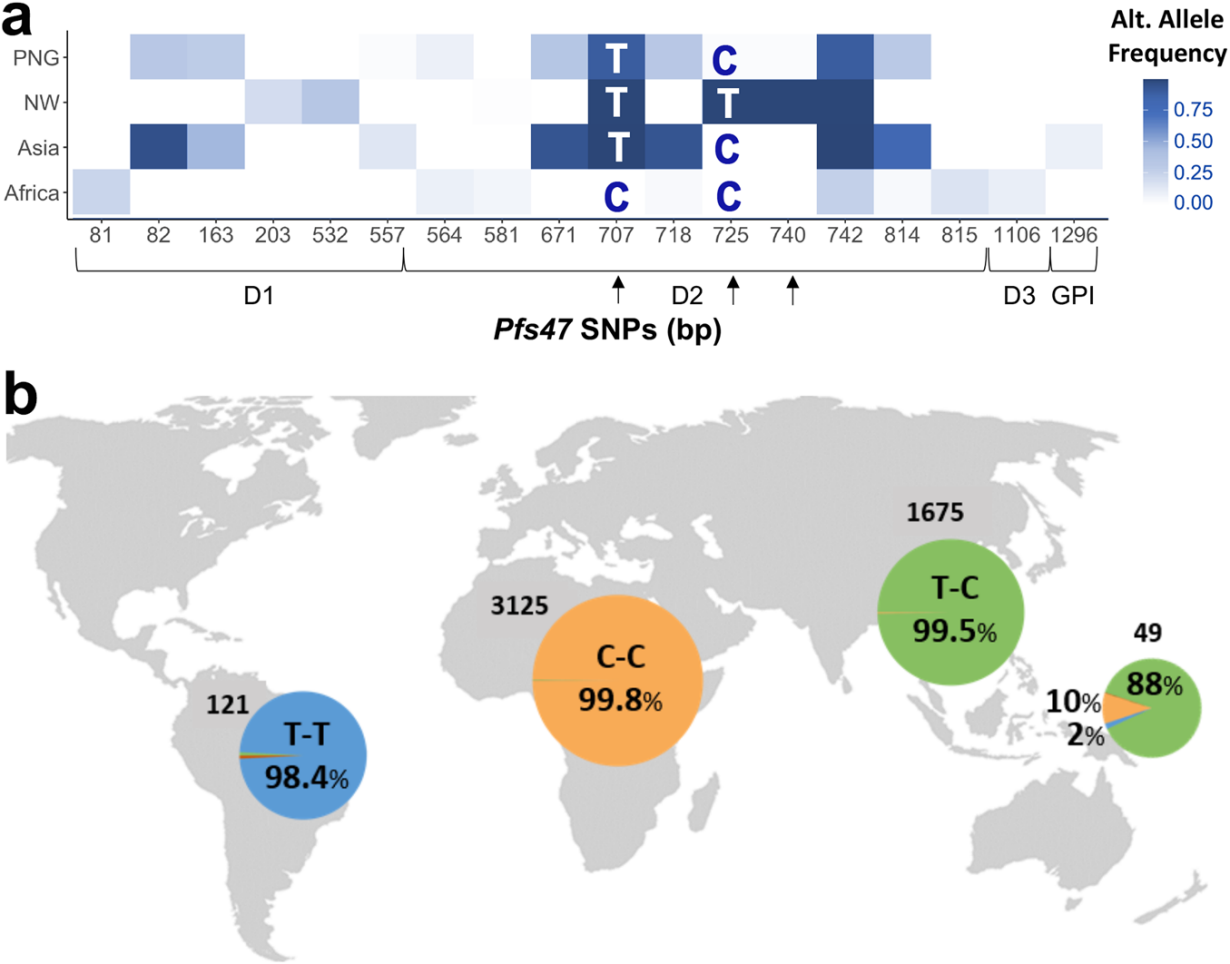
Alternate allele of *Pfs47* polymorphisms by geographic region. **a** Heat map of the frequency of 18 *Pfs47* SNPs with alternate allele frequencies >0.05 in Africa, Asia, the New World (NW), and Papua New Guinea (PNG)). The most common African Allele (GB4) was used as reference. *Pfs47* polymorphisms with highest differences between continents are indicated with arrows (SNPs at 707, 725, and 740 bp). The letters in the boxes indicate the most frequent *Pfs47* genotype combinations for SNPs 707 and 725 in each geographic region. Coding regions of predicted *Pfs47* Domain 1 (D1, 82–546 bp), Domain 2 (D2, 547–846 bp), Domain 3 (D3, 847–1239 bp), and GPI anchoring region (GPI, 1240–1317 bp) are indicated. **b** Frequency of the most common *Pfs47* genotype combinations for SNPs 707 and 725 by geographic region. The number of sequences analyzed per continent is indicated in the map. The dominant genotype(s) in each region is indicated by different colors, Africa (orange), Asia (green), New World (Blue). Papua New Guinea presents genotypes from different geographic regions.

A detailed analysis of 4,971 *Pfs47* DNA sequences from countries in different geographic regions (Africa, Asia, New World (NW), and Papua New Guinea (PNG)) (Supplementary Data 1) was conducted to determine the geographic population structure of *Pfs47*. A total of 84 *Pfs47* SNPs were identified (Supplementary Data 1), with 18 SNPs having an alternate allele frequency higher than 0.05 (Fig. 1a). *Pfs47* has three domains, with the central domain (Domain 2) being the most polymorphic, harboring 10 out of the 18 frequent SNPs present (Fig. 1a). The SNPs located at positions 707, 725, and 740 bp in Domain 2 have the most drastic differences in frequency between continents (Fig. 1a, Supplementary Data 1), and the genotype of SNP 740 appears linked to that of SNP 725 (Fig. 1a, Supplementary Data 1). The degree of population differentiation of *Pfs47* SNPs was established by determining Wright’s fixation index (*F*_ST_). Among all *Pfs47* polymorphisms, *Pfs47* SNP 707 presents the highest *F*_ST_ between populations from Africa and other continents (0.89–0.99), while *Pfs47* SNPs 725 and 740 present the highest *F*_ST_ between the New World and other continents (0.97–0.99) (Supplementary Data 2). Indeed, the combined genotype of SNPs 707 and 725 is sufficient to differentiate *Pfs47* haplotypes originating from African (C-C), Asian (T-C), or New World (T-T) origin with a probability of 99% (Table S1, Fig. 1a, b). Although the predominant genotype in Asia of SNPs 707 and 725 (T-C) is also the most frequent (0.88) in PNG, the most prevalent genotype from Africa (C-C) and the New World (T-T) are also present in PNG at lower frequencies, 0.10 and 0.02, respectively. While both SNPs 707 and 725 result in amino acid substitutions, it is interesting that the “C-T” combination for SNPs 707 and 725 was not detected in any continent.

**Fig. 2:**
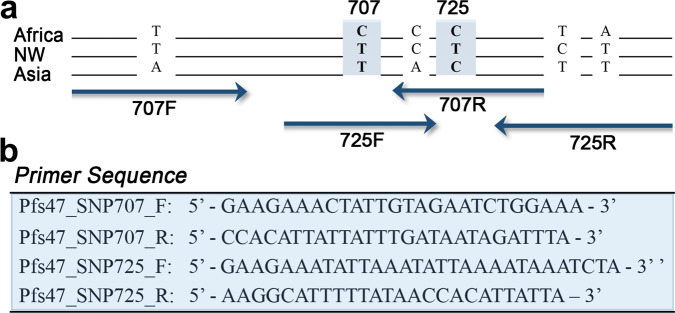
*Pfs47* polymorphisms near SNPs 707 and 725 and High-Resolution Melting assay (HRM) primer design. **a** Schematic representation of *Pfs47* polymorphisms in representative haplotypes of *P. falciparum* isolates from Africa (GB4), New World (7G8), and Asia (Asia 33). The diagram illustrates the approximate location of the forward and reverse primers for HRM assays to genotype SNPs at 707 and 725. **b** Primer sequences used in HRM assays.

**Fig. 3:**
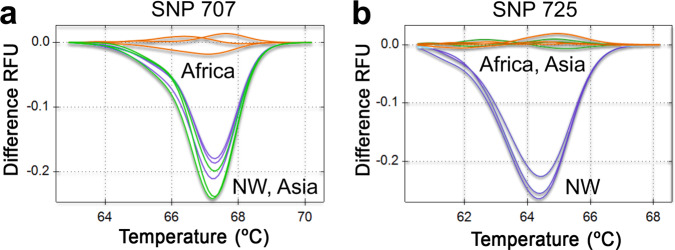
High-Resolution Melting (HRM) analysis of *Pfs47* SNPs 707 and 725. HRM profile in *P. falciparum* DNA samples from Africa (GB4, orange), Asia (Thai 17, green), and New World (7G8, blue) at three DNA concentrations (2, 0.2, 0.02 ng/$\upmu$l). **a** HRM difference curves for *Pfs47* SNP 707. **b** HRM difference curves for *Pfs47* SNP 725.

HRM assays were developed to facilitate genotyping of *Pfs47* SNPs 707 and 725. HRM analysis has been previously implemented and validated to genotype *Plasmodium* for molecular barcoding and detection of drug resistance^12–15^. The HRM assay consists of a PCR amplification, followed by careful melting curve analysis of the PCR products. This allows for fast genotyping of specific SNPs without the need for DNA sequencing^16^. Separate HRMs were necessary for each SNP, due to the presence of other non-informative polymorphisms in close proximity (Fig. 2a). Multiple potential primer sets were designed and tested, until two suitable primer sets (one pair for each SNP) for accurate HRM SNP assays were identified and optimized (Fig. 2b). The HRM assays were tested using two independent *P. falciparum* lines from each continent (Africa, Asia, and the New World). The HRM assay for *Pfs47* SNP 707 distinguished the predominant African allele from Asian and New World alleles with more than 96.6–99.8% clustering confidence (Figs. 3a, S1a, S2a, Table S2). Conversely, the HRM for *Pfs47* SNP 725 distinguished the main *Pfs47* New World allele from the African and Asian alleles with more than 97.2–99.6% clustering confidence (Figs. 3b, S1b, S2b, Table S3). The most frequent Asian *Pfs47* allele could be identified by analyzing the results from both HRM assays, since it clustered with the predominant New World allele in *Pfs47* SNP 707, and with the predominant African allele in *Pfs47* SNP 725 (Figs. 3a, b; S1a, b, S2a, b; Tables S2, S3). Both HRM assays were reproducible at template concentrations of 2, 0.2, and 0.02 ng/$\mu$l DNA (Figs. 3a, b; S1a, b; Tables S1, S2).

**Fig. 4:**
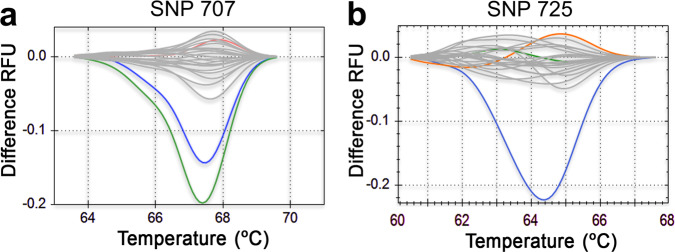
High-Resolution Melting (HRM) analysis of *Pfs47* SNPs 707 and 725 in field samples from Africa (gray). **a** HRM difference curves for *Pfs47* SNP 707 in 20 dry blood spots from Kalifabougou, Mali, Africa. **b** HRM difference curves for *Pfs47* SNP 725 in 20 dry blood spots from Kalifabougou, Mali, Africa. Reference sequences from Africa (GB4 orange), Asia (Thai 17, green), and New World (7G8 blue) are also included.

The robustness of the *Pfs47* HRM SNP assays to genotype clinical samples was established by testing dried blood spots collected from individuals presenting with acute febrile malaria in a cohort study in Kalifabougou, Mali that has been described previously^17^. Parasite densities in these blood samples (*n* = 20) ranged from 80,700 to 312,300 *P. falciparum* asexual parasites/μl of blood (Table S4). We were able to genotype *Pfs47* SNPs 707 and 725 in all samples using our HRM assays, with cycle threshold (C_T_) values ranging from 22.4 to 27.8 (Table S4) during the PCR amplifications. The HRM assays accurately identified in the Malian samples both *Pfs47* SNPs 707 and 725 from African origin, with more than 95% clustering confidence (Figs. 4a, b, S3, Table S5). The limit of detection of the *Pfs47* SNP 707 and 725 HRM assays was estimated by analyzing serial dilutions from three field blood samples with high (Sample 599), medium (Sample 309), or lower parasitemia (Sample 614). The *Pfs47* SNP 707 HRM was within the method recommended C_T_ value of 30 with accurate clustering up to an estimated parasitemia of 2,875 +/− 98 parasites/μl (Fig. S4a–d; Table S6), while the 725 HRM was within the method limit of detection up to 4,911+/− 136 parasites/ml (Fig. S5a–d; Table S7). It is worth noting that the detection limit of the *Pfs47* SNP HRM assays developed, could in principle be increased by analyzing a larger dried blood sample.

## Discussion

In countries with ongoing malaria elimination programs, monitoring and managing imported cases is critical to prevent the re-establishment of local disease transmission. For example, the Chinese national malaria surveillance and control system currently deploys costly vector control measures and extensive screening for *Plasmodium* infection in local residents in response to imported malaria cases^2^. Assessing the risk of local malaria transmission more accurately, by determining whether the imported parasite is compatible with the local mosquito vector population, would thus be helpful to determine the appropriate response.

Here, we validate the use of *Pfs47* SNPs as markers for assessing the geographic origin and transmission potential of a *P. falciparum* malaria case. Our analysis of an extensive number of *Pfs47* gene sequences (4,971) confirmed the strong population structure of this gene according to the continental origin of parasites^7^. In fact, *Pfs47* is one of the genes with the highest geographic population structure detected in the *P. falciparum* genome^10^, with *Pfs47* SNPs at 707, 725, and 740 bp having marked differences in allele frequency at the continent level (Fig. 1, Supplementary Data 1). We have previously shown that these SNPs are major determinants of the compatibility of different *Pfs47* haplotypes with distinct mosquito species from different continents^18,19^. This would suggest that the amino acids encoded by these *Pfs47* SNPs in Domain 2 are critical for the interaction of *Pfs47* with its mosquito midgut receptor^11^.

The presence of multiple *Pfs47* haplotypes typical of diverse geographic origins in PNG could be due to more diversity of local mosquito vectors, which allows for transmission of multiple *Pfs47* haplotypes. Alternatively, some anophelines in that region may be particularly permissive to malaria parasites with different *Pfs47* haplotypes. Additional information is necessary to understand the diverse *Pfs47* haplotypes in PNG. This includes more detailed information on the compatibility between parasites containing different *Pfs47* haplotypes with local vectors, as well as information on the distribution of *Pfs47* haplotypes circulating in these vectors across PNG. The multiple *Pfs47* haplotypes present in PNG could also complicate the identification of the geographic origin of malaria cases exported from this region. When available, travel information on the affected individual would establish if the case originated in PNG. Nevertheless, local anopheline mosquitoes from other continents presumably would also limit transmission of incompatible *Pfs47* haplotypes from PNG, which can be determined using the proposed HRM analysis.

In practical terms, if the *Pfs47* SNPs 707 and 725 genotype of a putative imported malaria case coincides with local genotype(s) of a pre-elimination malaria region, this would predict high potential for local transmission. Conversely, if the genotype of *Pfs47* SNPs 707 and 725 of a putative imported case differs from the local genotype(s), this would predict likely low potential for local transmission. In the latter case, *Pfs47* genotyping would also confirm that the malaria case is indeed imported and would provide its possible continental origin. The *Pfs47* HRM analysis presented here is not reliable for individuals co-infected with parasites from different continents (e.g., African and Asian *Pfs47* haplotypes combined). Boundary regions between continents, such as Northeast Africa and the Arabian Peninsula, could also be problematic if Asian vectors, such as *Anopheles stephensi*, co-exist with African anophelines.

If the *Pfs47* haplotypes in a patient sample do not cluster with the standards used in the HRM analysis we developed, it would be necessary to amplify and sequence the *Pfs47* Domain 2 region containing the most informative SNPs (707, 725, and 740 bp) to determine the geographic origin of the sample. Interestingly, the “C-T” combination for SNPs 707 and 725 was not detected in any continent, suggesting that it may code for an amino acid combination in Domain 2 of *Pfs47* that is deleterious to the parasite and is thus under strong negative selection.

*Plasmodium vivax Pvs47*, the orthologue of *Pfs47*, also presents a marked geographic population structure^20^, suggesting that *Pvs47* may also be used as a maker to assess geographic origin of *P. vivax*. However, the compatibility of *Pvs47* haplotypes with specific vectors remains to be determined. Thus, it is unclear if polymorphisms in *Pvs47* can be used to determine the risk of *P. vivax* importation and transmission.

Other genotyping assays have been used to determine the geographic origin of *P. falciparum*, but they typically require analyzing a large number of SNPs^21–23^. For example, an assay that identifies the continent of origin of malaria cases with 92% accuracy uses 23 mitochondrial and apicoplast SNPs^23^. However, genotyping only 2 SNPs using the *Pfs47* HRM assays is a much simpler strategy. In addition, previous SNP-based assays to determine the geographic origin of *P. falciparum* are limited in that their markers were not biologically linked to transmission risk. The relatively simple and low-cost HRM *Pfs47* genotyping assays developed here, provide a practical epidemiological tool to establish the putative geographic origin of imported *P. falciparum* malaria cases and to assess transmission risk in a particular region.

## Methods

### *Pfs47* sequences and statistical analysis

*Pfs47* gene sequences were obtained from the literature^7,24^ and recovered from *P. falciparum* samples in the publicly available databases of the Malaria Genomic Epidemiology Network^25^ (MalariaGEN). A total of 4,971 *Pfs47* haplotype sequences were used, originating from Africa (3,126), New World (121), Asia (1,675), and Papua New Guinea (PNG, 49). The New World was the continent least represented in the analysis of *Pfs47* sequences. This publication uses data from the MalariaGEN *P. falciparum* Community Project, PfCP (www.malariagen.net/projects/p-falciparum-community-project) and the Pf3K project (2016) pilot data release 5 (www.malariagen.net/data/pf3k-5). Genome sequencing was performed by the Wellcome Trust Sanger Institute and the Community Projects is coordinated by the MalariaGEN Resource Centre with funding from the Wellcome Trust (098051, 090770). The *Pfs47* sequences from Pf3K polygenomic samples were obtained after deconvolution using DEploid as described^26^. In the case of the *Pfs47* sequences in samples from other sources, only the major allele was analyzed as described^24^. Single nucleotide polymorphisms (SNPs) in the recovered *Pfs47* sequences were analyzed using dnaSP version 6^27^ according to their geographic allele occurrence. The most frequent African allele (GB4) was used as a reference sequence.

### *Plasmodium falciparum* samples

Laboratory maintained *P. falciparum* lines from Africa (NF54, GB4), New World (7G8, HB3), and Asia (Thai 17, P95-15) were used. *P. falciparum*-infected dried blood spots (13-mm diameter) were collected in 2013 with informed consent from subjects (*n* = 20) enrolled in a cohort study in Kalifabougou, Mali, who presented with acute febrile malaria. The cohort has been described in detail previously^17^. Blood spots were collected from finger prick samples onto Whatman^®^ protein saver cards, dried, and stored at room temperature in individual sealed bags until their use. From the same finger prick blood sample, thick blood smears were stained with Giemsa and counted against 300 leukocytes, and *P. falciparum* densities were recorded as the number of asexual parasites per µL of whole blood based on a mean leukocyte count of 7500 cells per µl. Each smear was evaluated separately by at least 2 expert microscopists.

### DNA extractions

DNA was extracted from *P. falciparum* cultures using the QIAamp® DNA Mini Kit (Qiagen). Briefly, saponin pellets from *P. falciparum* cultures were used to extract DNA. In the case of the dried blood spots, one-third of a 13-mm spot was used for DNA extraction. During optimization of the HRM, it was determined that the elution of DNA should be done in AE buffer (Qiagen) diluted 1 to 10 in nuclease-free water, since higher concentrations of the AE buffer affected the reproducibility of the HRM assay. The DNA samples used for geographic origin standards in the HRM assay were extracted from dried blood spots, using the same filter paper as the field-collected samples. Once all DNA had been extracted from standards, each was brought to a concentration of 2 ng/µl in 1:10 Buffer AE. Ten-fold serial dilutions were done in 1:10 Buffer AE, with final concentrations at 2, 0.2, and 0.02 ng/µl.

### SNP assay primer design, qPCR, and HRM assay

Several sets of primers were designed due to the highly polymorphic nature of *Pfs47* Domain 2, where both SNPs 707 and 725 are located (Fig. 2a). These primers were tested with all samples (GB4, NF54, 7G8, HB3, Thai17, P95-15, and Malian Kali), and ultimately primers were chosen based on their efficiency and reproducibility with all samples, as reflected by the lowest Ct values, and single peak in the Melting Curves and accurate HRM analysis. The primers used for SNP 707 bp were Pfs47_SNP707_F: GAAGAAACTATTGTAGAATCTGGAAA, Pfs47_SNP707_R: CCACATTATTATTTGATAATAGATTTA, and the primers for SNP 725 bp were Pfs47SNP725_F: GAAGAAATATTAAATATTAAAATAAATCTA, and Pfs47SNP725_R: AAGGCATTTTTATAACCACATTATTA (Fig. 2b). SNP 707 and 725 bp reactions were run separately. Primers were diluted first in TE to 100 μM, and then in nuclease-free water to 8 µM. Each reaction was 20 µL total volume: 10 µL of Precision Melt Supermix (Bio-Rad), 4 µl nuclease-free water, 1 µL of each 8 µM primer (forward and reverse), and 4 µL of template DNA. qPCR Conditions were (1) 95 °C for 5 min, (2) 95 °C for 10 s, (3) 50 °C for 30 s, (4) 60 °C for 30 s + plate read, (5) go back to step 2 and repeat 39 more times, (6) 95 °C for 30 s, (7) 55 °C for 1 min, (8) Melt Curve 60–90 °C at 0.2 °C increments for 10 s including a plate read. All samples were run in duplicates. Analysis of the HRM assay was done using Precision Melt Analysis Software version 1.3 (Bio-Rad).

### *Pfs47* SNP 707 and 725 HRM detection limit

The detection limit was defined as the lowest parasitemia in the blood samples that would generate reproducible HRM signal. According to the HRM reagent kit used (Bio-Rad), reliable results are obtained with samples that reach the signal threshold before 30 PCR cycles (30Ct).

### Statistics and reproducibility

The reproducibility of each HRM genotype was assessed running each sample at least in duplicate. The reproducibility of HRM genotypes corresponding to a given geographical area was assessed by running at least two biological samples from a given region.

### Ethics statement

The ethics committee of the Faculty of Medicine, Pharmacy and Dentistry at the University of Sciences, Techniques and Technology of Bamako, and the Institutional Review Board of NIAID, NIH approved the Mali study (NIH protocol number 11-I-N126; ClinicalTrials.gov NCT01322581). Written, informed consent was obtained from the parents or guardians of participating children or from adult participants.

### Reporting summary

Further information on research design is available in the Nature Research Reporting Summary linked to this article.

## Supplementary information

Supplementary Information

Description of Additional Supplementary Files

Supplementary Data 1

Supplementary Data 2

Supplementary Data 3

Reporting Summary

## Data Availability

*Pfs47* gene sequences were retrieved from the literature^7,24^ and from the publicly available databases of the Malaria Genomic Epidemiology Network^25^ (MalariaGEN) *P. falciparum* Community Project, PfCP (www.malariagen.net/projects/p-falciparum-community-project), and the Pf3K project (2016) pilot data release 5 (www.malariagen.net/data/pf3k-5). Sample ID, geographic origin, source, study, and accession numbers are indicated in Supplementary Data 3.
