# Supplementary material for: A genotyping assay to determine geographic origin and transmission potential of Plasmodium falciparum malaria cases
Source: Commun Biol. 2021 Sep 30;4:1145. doi: 10.1038/s42003-021-02667-0 (PMC8484479; doi:10.1038/s42003-021-02667-0)
Supplement: Supplementary file 1 — Supplementary Information [file 42003_2021_2667_MOESM1_ESM.pdf]

Supplementary Information

A Genotyping Assay to Determine Geographic Origin and Transmission Potential of *Plasmodium falciparum* malaria cases

Alvaro Molina-Cruz[1]*, Nadia Raytselis[1], Roxanne Withers[1], Ankit Dwivedi[2], Peter D. Crompton[3], Boubacar Traore[4], Giovanna Carpi[5], Joana C. Silva[2,6], Carolina Barillas-Mury[1]*

[1] Laboratory of Malaria and Vector Research, National Institute of Allergy and Infectious Diseases, National Institutes of Health, Rockville, MD, USA 20852

[2] Institute for Genome Sciences, University of Maryland School of Medicine, Baltimore, MD, 21201, USA

[3] Malaria Infection Biology and Immunity Section, Laboratory of Immunogenetics, National Institute of Allergy and Infectious Diseases, NIH, Rockville, MD 20852, USA

[4] Mali International Center of Excellence in Research, University of Sciences, Techniques and Technologies of Bamako, Bamako, Mali

[5] Department of Biological Sciences, Purdue University, West Lafayette, IN, 47907, USA

[6] Department of Microbiology and Immunology, University of Maryland School of Medicine, Baltimore, MD, 21201, USA

*To whom correspondence should be addressed: amolina-cruz@niaid.nih.gov; cbarillas@niaid.nih.gov

Table S1. Frequency of Pfs47 genotypes in SNPs at 707 and 725 bp, in different geographic regions. The frequency of *Pfs47* genotypes in 707 and 725 bp SNPs were determined in 4,971 gene sequences from different regions (Africa, Asia, New World (NW), and Papua New Guinea (PNG)). The total number of sequences analyzed from each region is indicated.

**Pfs47 SNP Genotype**	**Genotype frequency by Region (n)**			
**707…725**	**Africa (3125)**	**Asia (1675)**	**New World (121)**	**PNG (49)**
C…C	0.9984	0.0054	0.0083	0.1020
T…C	0.0013	0.9946	0	0.8776
T…T	0.0003	0	0.9917	0.0204
C…T	0	0	0	0

Table S2. PCR efficiency and HRM clustering of Pfs47 genotypes of SNP 707 in reference sequences of *P. falciparum* parasites originated in Africa (orange), New World (blue), and Asia (green). DNA template concentrations used were 2, 0.2, and 0.002 (ng/µl).

Sample	PCR Efficiency (%)	Conc. (ng/ul)	Rep. #1		Rep. #2	
			Cluster	Conf. (%)	Cluster	Conf. (%)
NF54	95.2	2	2	98.7	2	99.5
NF54		0.2	2	99.6	2	99.4
NF54		0.02	2	98.2	2	98.4
GB4	97.8	2	2	99.4	2	99.0
GB4		0.2	2	99.6	2	99.8
GB4		0.02	2	98.8	2	98.7
7G8	89.9	2	1	99.1	1	99.2
7G8		0.2	1	98.9	1	99.3
7G8		0.02	1	99.6	1	99.7
HB3	96.0	2	1	97.7	1	98.0
HB3		0.2	1	97.1	1	98.1
HB3		0.02	1	99.3	1	98.9
Thai 17	96.0	2	1	98.3	1	99.6
Thai 17		0.2	1	99.5	1	98.4
Thai 17		0.02	1	96.6	1	98.3
P95-15	92.5	2	1	99.7	1	99.7
P95-15		0.2	1	99.8	1	99.2
P95-15		0.02	1	97.7	1	97.4

Table S3. PCR efficiency and HRM clustering of Pfs47 genotype of SNP 725 in reference sequences of P. falciparum parasites originated in Africa (orange), New World (blue), and Asia (green). DNA template concentrations used were 2, 0.2 and 0.002 (ng/µl).

			Rep. #1		Rep. #2	
**Sample**	**PCR Reaction Efficiency (%)**	**Conc. (ng/ul)**	**Cluster**	**Conf. (%)**	**Cluster**	**Conf. (%)**
NF54	99.6	2	1	98.1	1	98.3
NF54		0.2	1	99.4	1	98.8
NF54		0.02	1	98.7	1	99.7
GB4	95.3	2	1	98.8	1	96.9
GB4		0.2	1	97.6	1	99.3
GB4		0.02	1	99.4	1	98.3
7G8	95.2	2	2	98.6	2	99.2
7G8		0.2	2	97.3	2	98.4
7G8		0.02	2	98.1	2	97.7
HB3	95.8	2	2	98.1	2	98.7
HB3		0.2	2	97.8	2	98.0
HB3		0.02	2	97.2	2	96.6
Thai 17	89.4	2	1	98.6	1	98.3
Thai 17		0.2	1	98.7	1	98.9
Thai 17		0.02	1	99.6	1	98.8
P95-15	98.6	2	1	97.7	1	98.0
P95-15		0.2	1	98.6	3	98.0
P95-15		0.02	1	98.4	1	99.2

Table S4. Parasitemia and Ct values of *Pfs47* HRM for SNPs 707 and 725, of field samples from Mali, Africa.

**qPCR Parameters for Infected Kalifabougou, Mali, Africa Samples**			
Sample ID	Parasite Density (parasites/ul)	*Pfs47* SNP 707 Ct values (mean +/- SE)	*Pfs47* SNP 725 Ct values (mean +/- SE)
Kali 599	312,300	23.16 +/- 0.08	23.37 +/- 0.03
Kali 589	252,900	22.44 +/- 0.02	22.61 +/- 0.04
Kali 583	243,600	25.11 +/- 0.03	25.21 +/- 0.05
Kali 604	196,000	24.97 +/- 0.16	25.22 +/- 0.00
Kali 680	173,100	23.82 +/- 0.02	23.83 +/-0.10
Kali 309	136,500	24.64 +/- 0.06	24.83 +/- 0.04
Kali 241	131,850	25.39 +/- 0.09	25.74 +/- 0.06
Kali 687	130,500	25.43 +/- 0.01	25.70 +/- 0.03
Kali 525	122,250	24.44 +/- 0.10	24.39 +/-0.05
Kali 629	117,750	25.44 +/- 0.05	25.31 +/- 0.06
Kali 471	116,700	26.50 +/- 0.16	26.40 +/-0.02
Kali 625	105,000	25.35 +/- 0.08	25.24 +/- 0.02
Kali 445	104,850	24.22 +/- 0.03	24.10 +/- 0.00
Kali 460	102,750	26.37 +/- 0.04	26.41 +/- 0.01
Kali 606	95,250	27.48 +/- 0.04	27.69 +/- 0.03
Kali 614	93,900	25.10 +/- 0.03	25.32 +/- 0.04
Kali 694	91,650	28.77 +/- 0.19	28.80 +/- 0.03
Kali 399	89,850	26.72 +/- 0.02	26.59 +/- 0.01
Kali 502	82,050	27.80 +/- 0.07	27.92 +/- 0.01
Kali 691	80,700	26.77 +/- 0.22	26.91 +/-0.01

Table S5. HRM analsyis of *Pfs47* SNPs 707 and 725 in field samples from Africa. HRM clustering of Pfs47 genotypes at SNPs 707 and 725 in reference sequences of *P. falciparum* parasites originated in Asia (green), New World (blue), Africa (orange) and in field samples from Kalifabougou, Mali, Africa. *Pfs47* haplotype standards were run at 0.2ng/µl). All samples were run in duplicate.

Pfs47 SNP 707

Sample	Cluster	%Conf.
Kali 599	1	98.2
Kali 599	1	99.7
Kali 589	1	99.8
Kali 589	1	99.0
Kali 583	1	98.8
Kali 583	1	99.0
Kali 604	1	99.4
Kali 604	1	99.8
Kali 680	1	99.4
Kali 680	1	99.2
Kali 680	1	99.4
Kali 680	1	99.2
Kali 309	1	99.2
Kali 309	1	99.3
Kali 241	1	99.5
Kali 241	1	99.7
Kali 687	1	99.8
Kali 687	1	99.9
Kali 525	1	99.3
Kali 525	1	99.6
Kali 629	1	99.7
Kali 629	1	99.5
Kali 471	1	98.9
Kali 471	1	98.7
Kali 625	1	99.7
Kali 625	1	99.4
Kali 445	1	97.8
Kali 445	1	99.9
Kali 460	1	99.3
Kali 460	1	99.5
Kali 614	1	99.9
Kali 614	1	99.7
Kali 694	1	99.1
Kali 694	1	99.3
Kali 399	1	99.4
Kali 399	1	97.7
Kali 502	1	98.7
Kali 502	1	98.8
Kali 691	1	99.5
Kali 691	1	99.3
NF54	1	99.5
NF54	1	99.4
GB4	1	99.7
GB4	1	98.9
Thai 17	2	99.9
Thai 17	2	99.9
P95-15	2	99.9
P95-15	2	99.8
7G8	2	99.2
7G8	2	99.1
HB3	2	97.3
HB3	2	91.6

Pfs47 SNP 725

Sample	Cluster	%Conf.
Kali 599	1	98.1
Kali 599	1	97.5
Kali 589	1	98.0
Kali 589	1	94.0
Kali 583	1	97.0
Kali 583	1	97.6
Kali 604	1	98.7
Kali 604	1	97.8
Kali 680	1	98.7
Kali 680	1	98.3
Kali 309	1	98.2
Kali 309	1	98.2
Kali 241	1	99.0
Kali 241	1	94.7
Kali 687	1	97.7
Kali 687	1	97.7
Kali 525	1	97.9
Kali 525	1	97.9
Kali 629	1	98.5
Kali 629	1	98.3
Kali 471	1	99.4
Kali 471	1	99.6
Kali 625	1	98.1
Kali 625	1	98.2
Kali 445	1	97.4
Kali 445	1	98.0
Kali 460	1	99.7
Kali 460	1	99.8
Kali 606	1	89.2
Kali 606	1	97.2
Kali 614	1	99.5
Kali 614	1	99.2
Kali 694	1	99.5
Kali 694	1	99.1
Kali 399	1	98.8
Kali 399	1	98.8
Kali 502	1	97.9
Kali 502	1	99.8
Kali 691	1	98.6
Kali 691	1	97.7
NF54	1	97.9
NF54	1	98.0
GB4	1	96.4
GB4	1	97.1
Thai 17	1	97.8
Thai 17	1	98.3
P95-15	1	97.9
P95-15	1	98.5
7G8	2	98.7
7G8	2	99.1
HB3	2	98.0
HB3	2	98.8

Table S6. Pfs47 SNP 707 HRM detection limit analysis and PCR efficiency. Malaria field samples from Kalifaboubou, Mali, Africa, were analyzed at serial dilutions to estimate the lowest parasitemia that would produce accurate and reproducible clustering.

***Pfs47* SNP 707: Kalifabougou, Mali, Africa Samples**				
Sample	**PCR Efficiency (%)**	Cluster	% Confidence	Ct
Kali 599 1:1	93.7	1	98.5	22.71
1:1		1	98.8	22.92
1:10		1	98.1	26.16
1:10		1	98.2	26.19
1:100		1	98.2	29.76
1:100		1	98.5	29.80
Kali 309 1:1	90.1	1	98.4	24.05
1:1		1	98.2	24.20
1:10		1	98.6	27.55
1:10		1	98.5	27.46
1:100		1	97.9	31.19
1:100		1	97.3	31.39
Kali 614 1:1	91.9	1	98.5	24.71
1:1		1	97.5	24.71
1:10		1	98.4	28.13
1:10		1	97.9	28.32
1:100		1	98.0	31.57
1:100		2	88.9	31.99
NF54 0.2ng/uL		1	98.1	29.50
NF54 0.2ng/uL		1	99.5	29.34
HB3 0/2ng/uL		3	97.1	23.67
HB3 0/2ng/uL		3	98.4	23.67
Thai17 0.2ng/uL		3	99.1	26.03
Thai17 0.2ng/uL		3	94.6	26.01

Table S7. Pfs47 SNP 725 HRM detection limit analysis and PCR efficiency. Malaria field samples from Kalifaboubou, Mali, Africa, were analyzed at serial dilutions to estimate the lowest parasitemia that would produce accurate and reproducible clustering.

Pfs47 SNP 707: Kalifabougou, Mali, Africa Samples				
Sample	**PCR Efficiency (%)**	Cluster	% Confidence	Ct
Kali 599 1:1	100	1	89.9	24.69
1:1		1	92.7	23.64
1:10		1	96.7	27.18
1:10		1	97.1	27.10
1:100		1	97.1	30.55
1:100		1	97.9	30.81
Kali 309 1:1	99.7	1	95.0	25.13
1:1		1	92.1	25.15
1:10		1	96.5	28.49
1:10		1	96.8	28.28
1:100		1	96.9	31.84
1:100		1	61.2	31.76
Kali 614 1:1	97.1	1	94.0	25.66
1:1		1	86.2	25.79
1:10		1	97.4	29.14
1:10		1	97.6	29.21
1:100		1	97.9	32.39
1:100		1	97.0	32.62
NF54 0.2ng/uL		1	95.2	30.31
NF54 0.2ng/uL		1	97.7	30.39
HB3 0/2ng/uL		2	97.1	24.20
HB3 0/2ng/uL		2	98.4	24.40
Thai17 0.2ng/uL		1	90.9	27.20
Thai17 0.2ng/uL		1	98.0	27.09

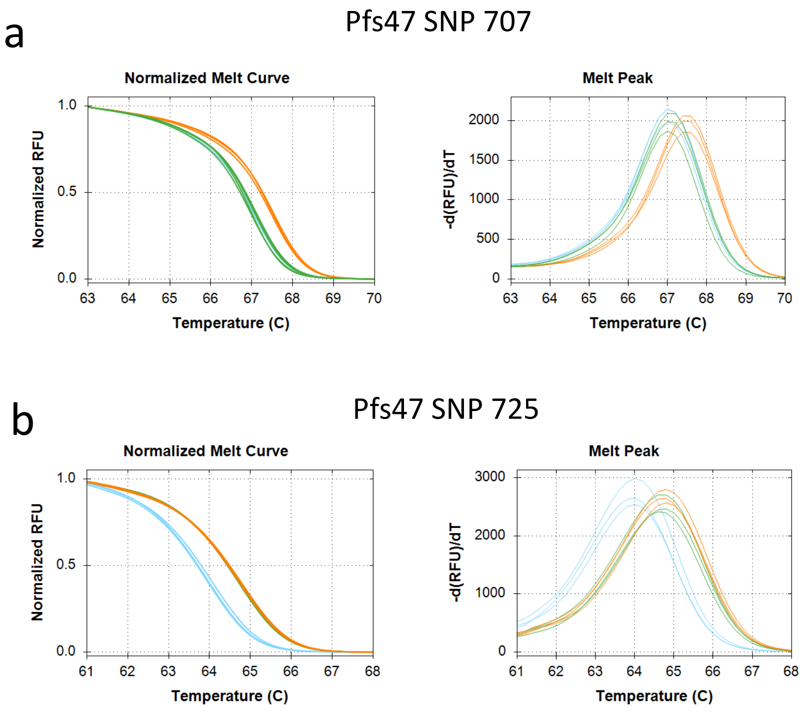

Fig S1. HRM analysis of *Pfs47* SNPs 707 and 725 in reference sequences from Africa (GB4; orange), Asia (Thai 17; green) and New World (7G8; blue) at 3 concentrations (2,0.2,0.02ng/µl). The melting curve and Tm point analysis are shown for (a) *Pfs47* SNP 707 and for (b)*Pfs47* SNP 725.

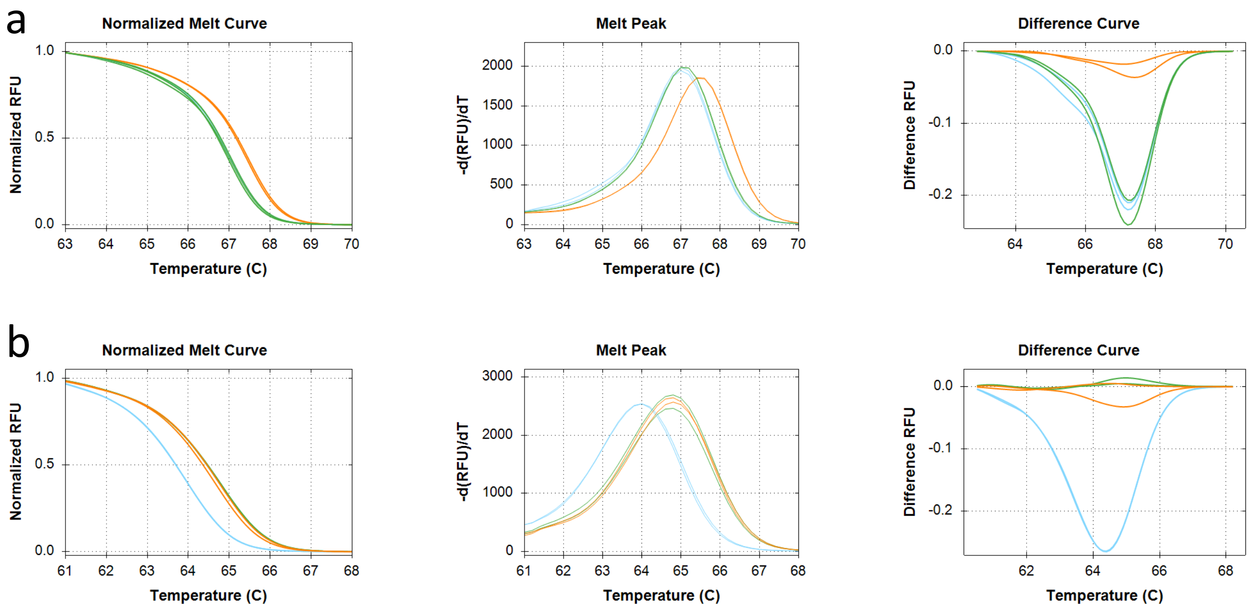

Fig S2. HRM analysis of Pfs47 SNPs 707 and 725 in reference sequences from Africa (GB4, NF54; orange), Asia (Thai 17, P95; green) and New World (7G8, HB3; blue) at 2.0 ng/µl. The melting curve, Tm analysis, and HRM difference curve, are shown for *Pfs47* SNP 707 (a) and for Pfs47 SNP 725 (b).

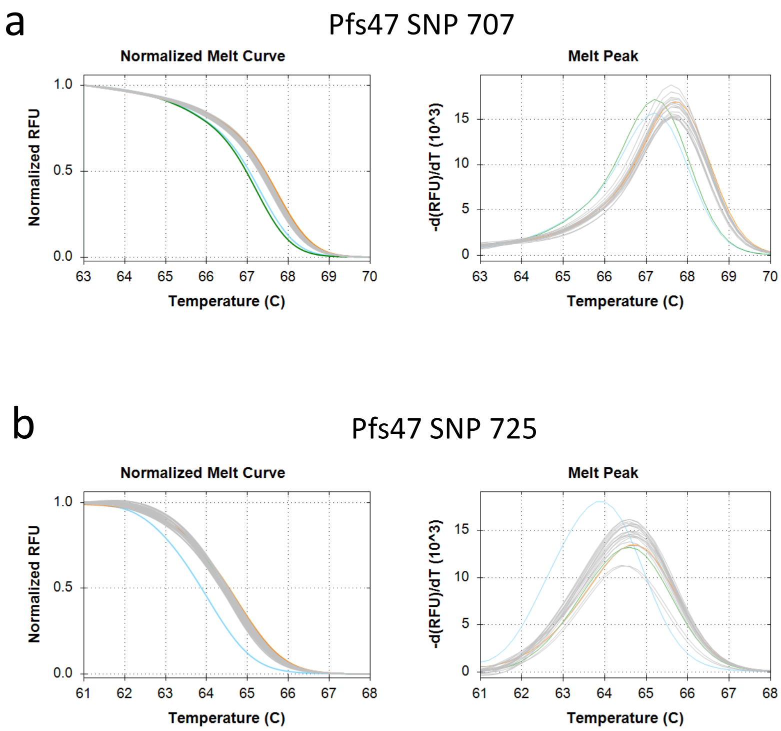

Fig S3. HRM analysis of *Pfs47* SNPs 707 and 725 in field samples from Kalifabougou, Mali, Africa. Also included are reference sequences from Africa (GB4 orange), Asia (Thai 17, green) and New World (7G8 blue). The melting curve, Tm analysis, and HRM difference curve are shown for Pfs47 SNP 707 (a) and for Pfs47 SNP 725 (b). Field samples from Mali appear in gray.

a

SNP 707- Kali 599	
CT	Parasites/µl
22.71	312,300
22.92	312,300
26.16	31,230
26.18	31,230
29.76	3,123
29.80	3,123

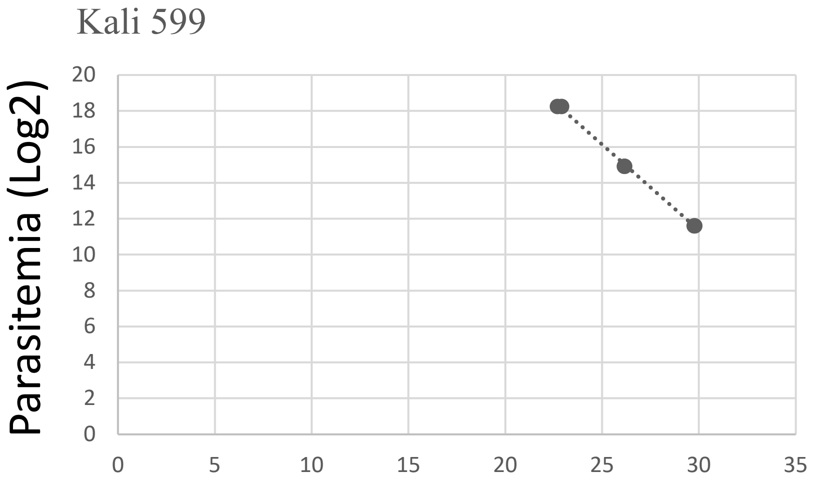

b

SNP 707- Kali 309	
CT	Parasites/µl
24.05	136,500
24.20	136,500
27.55	13,650
27.46	13,650
31.19	1,365
31.39	1,365

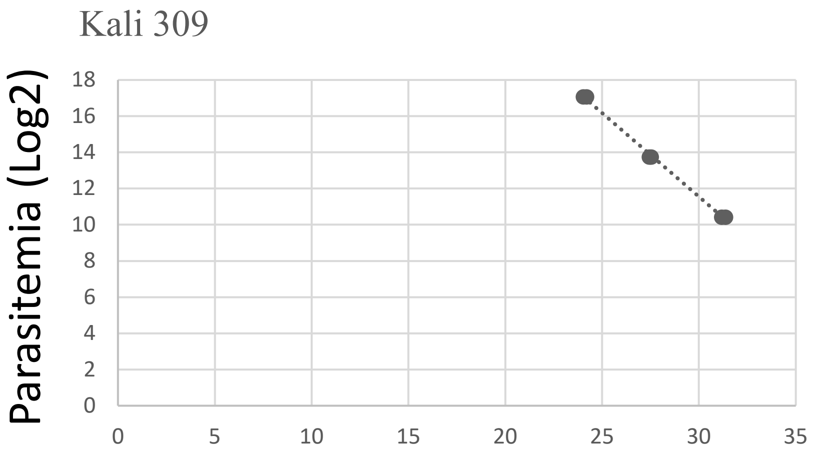

c

SNP 707- Kali 614	
CT	Parasites/µl
24.71	93,900
24.71	93,900
28.13	13.20
28.32	13.20
31.57	9.87
31.99	9.87

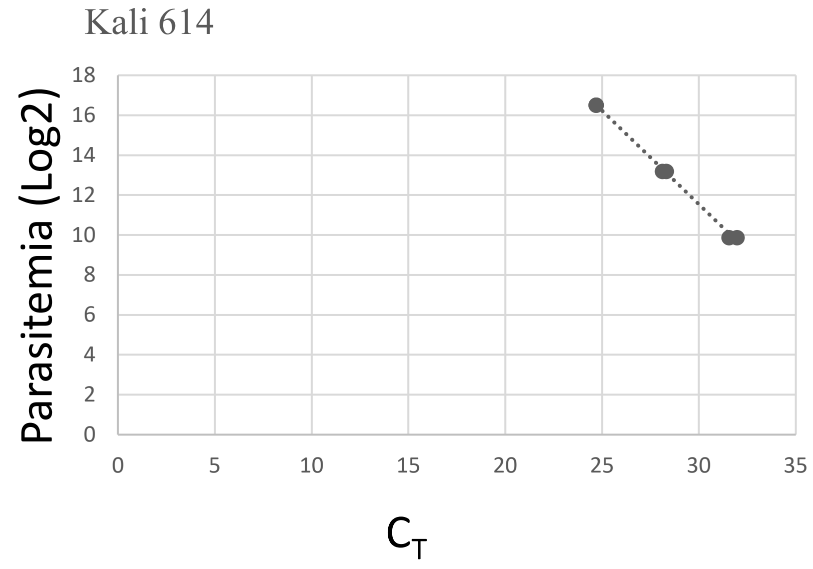

d

**SNP 707**	
**Sample**	**Parasites/µl at 30C_T**
599	2634.37
614	3005.74
309	2986.16
Average +/- SE	2875.42+/-98.52

Fig S4. *Pfs47* SNP 707 HRM detection limit analysis. Three malaria field samples from Kalifabougou Mali, Africa (Kali 599 (a), 309 (b), 614 (c)), were analyzed at 10-fold serial dilutions to estimate, by linear regression analysis, the lowest parasitemia that would produce a C_T of 30 (d), the recommended limit of the HRM method used (BioRad). HRM assays were done in duplicates.

a

SNP 725- Kali 599	
CT	Parasites/µl
24.69	312,300
23.64	312,300
27.18	31,230
27.10	31,230
30.55	3,123
30.81	3,123

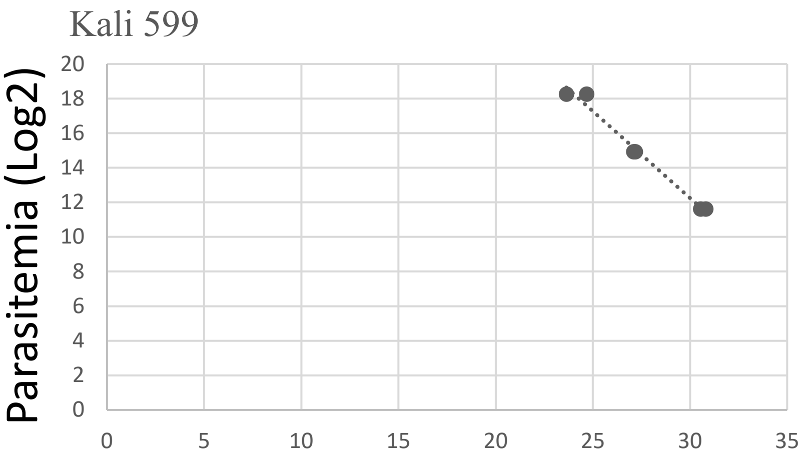

b

SNP 725- Kali 309	
CT	Parasites/µl
25.13	136,500
25.15	136,500
28.49	13,650
28.28	13,650
31.84	1,365
31.76	1,365

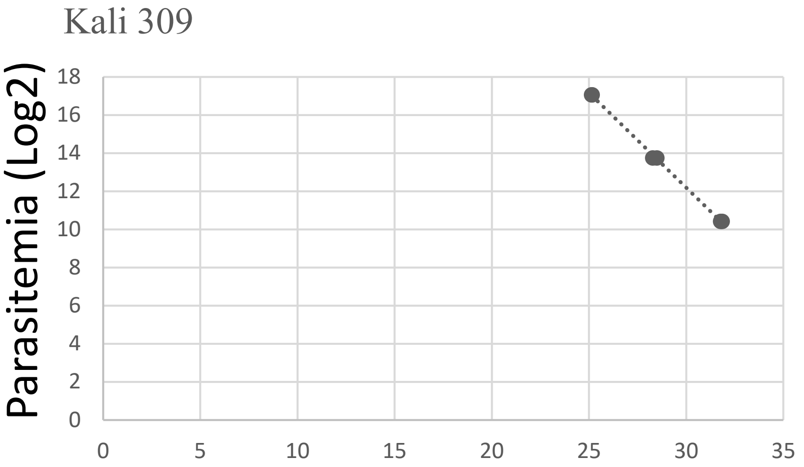

c

SNP 725- Kali 614	
CT	Parasites/µl
25.66	93,900
25.79	93,900
29.14	13.20
29.21	13.20
32.39	9.87
32.62	9.87

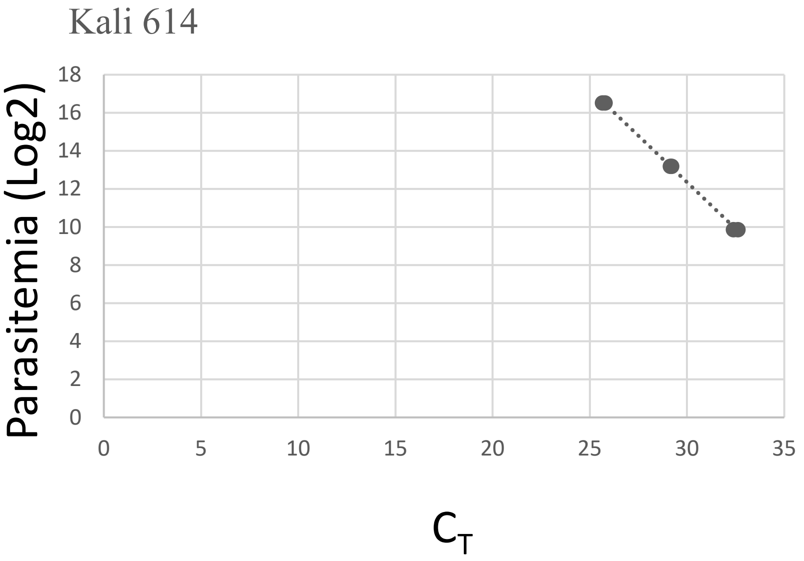

d

**SNP 725**	
**Sample**	**Parasites/µl at 30C_T**
599	4872.70
614	4644.38
309	5218.85
Average +/- SE	4911.98+/-136.35

Fig S5. *Pfs47* SNP 725 HRM detection limit analysis. Three malaria field samples from Kalifabougou, Mali, Africa (Kali 599 (a), 309 (b), 614 (c)), , were analyzed at 10-fold serial dilutions to estimate the lowest parasitemia that would produce a CT under 30 (d), the recommended limit of the HRM method used (BioRad). HRM assays were done in duplicates
