# Supplementary material for: A genotyping assay to determine geographic origin and transmission potential of Plasmodium falciparum malaria cases
Source: Commun Biol. 2021 Sep 30;4:1145. doi: 10.1038/s42003-021-02667-0 (PMC8484479; doi:10.1038/s42003-021-02667-0)
Supplement: Supplementary file 2 — Description of Additional Supplementary Files [file 42003_2021_2667_MOESM2_ESM.pdf]

Description of Additional Supplementary Files

File name: Supplementary Data 1.
Description: Geographic origin of *Plasmodium falciparum* Pfs47 sequences analyzed and Single Nucleotide Polymorphism (SNP) altern allele frequency.

File name: Supplementary Data 2.
Description: Fixation index (F_{ST}) of Pfs47 Single Nucleotide Polymorphisms between geographic regions.

File name: Supplementary Data 3.
Description: Source and ID of *Plasmodium falciparum* samples analyzed.
