# Supplementary material for: A genotyping assay to determine geographic origin and transmission potential of Plasmodium falciparum malaria cases
Source: Commun Biol. 2021 Sep 30;4:1145. doi: 10.1038/s42003-021-02667-0 (PMC8484479; doi:10.1038/s42003-021-02667-0)
Supplement: Supplementary file 6 — Reporting Summary [file 42003_2021_2667_MOESM6_ESM.pdf]

nature portfolio

Corresponding author(s): Carolina Barillas-Mury, Alvaro Molina-Cruz

Last updated by author(s): Aug 9, 2021

Reporting Summary

Nature Portfolio wishes to improve the reproducibility of the work that we publish. This form provides structure for consistency and transparency in reporting. For further information on Nature Portfolio policies, see our Editorial Policies and the Editorial Policy Checklist.

Statistics

For all statistical analyses, confirm that the following items are present in the figure legend, table legend, main text, or Methods section.

n/a	Confirmed	
☐	☒	The exact sample size (*n*) for each experimental group/condition, given as a discrete number and unit of measurement
☐	☒	A statement on whether measurements were taken from distinct samples or whether the same sample was measured repeatedly
☐	☒	The statistical test(s) used AND whether they are one- or two-sided<br>*Only common tests should be described solely by name; describe more complex techniques in the Methods section.*
☒	☐	A description of all covariates tested
☒	☐	A description of any assumptions or corrections, such as tests of normality and adjustment for multiple comparisons
☒	☐	A full description of the statistical parameters including central tendency (e.g. means) or other basic estimates (e.g. regression coefficient) AND variation (e.g. standard deviation) or associated estimates of uncertainty (e.g. confidence intervals)
☒	☐	For null hypothesis testing, the test statistic (e.g. *F*, *t*, *r*) with confidence intervals, effect sizes, degrees of freedom and *P* value noted<br>*Give P values as exact values whenever suitable.*
☒	☐	For Bayesian analysis, information on the choice of priors and Markov chain Monte Carlo settings
☒	☐	For hierarchical and complex designs, identification of the appropriate level for tests and full reporting of outcomes
☒	☐	Estimates of effect sizes (e.g. Cohen's *d*, Pearson's *r*), indicating how they were calculated

Our web collection on statistics for biologists contains articles on many of the points above.

Software and code

Policy information about availability of computer code

Data collection	The qPCR and melting point were collected with CFX Maestro 1.1 (BioRad).
Data analysis	The High Resolution Melting analysis was done with Precision Melt Analysis Software version 1.3 (BioRad).

For manuscripts utilizing custom algorithms or software that are central to the research but not yet described in published literature, software must be made available to editors and reviewers. We strongly encourage code deposition in a community repository (e.g. GitHub). See the Nature Portfolio guidelines for submitting code & software for further information.

Data

Policy information about availability of data

All manuscripts must include a data availability statement. This statement should provide the following information, where applicable:

- Accession codes, unique identifiers, or web links for publicly available datasets
- A description of any restrictions on data availability
- For clinical datasets or third party data, please ensure that the statement adheres to our policy

Pfs47 gene sequences were retrieved from the literature8,25 and from the publicly available databases of the Malaria Genomic Epidemiology Network26 (MalariaGEN) P. falciparum Community Project, PfCP (www.malariagen.net/projects/p-falciparum-community-project), and the Pf3K project (2016) pilot data release 5 (www.malariagen.net/data/pf3k-5). Sample ID, geographic origin, source, study and accession numbers are indicated in Supplementary Data 3.

Field-specific reporting

Please select the one below that is the best fit for your research. If you are not sure, read the appropriate sections before making your selection.

☒ Life sciences ☐ Behavioural & social sciences ☐ Ecological, evolutionary & environmental sciences

For a reference copy of the document with all sections, see nature.com/documents/nr-reporting-summary-flat.pdf

Life sciences study design

All studies must disclose on these points even when the disclosure is negative.

Sample size

The BioRad software used to for the HRM analysis is proprietary and provides confidence levels based on confidence intervals. Due to its proprietary status, we were not able to obtain information from BioRad regarding the statistical methods used. We consulted with a biostatistician, and he concluded that without this information it is not possible to establish a power test. However, in this manuscript, our goal when analyzing field samples was to determine whether the assay that was established with cultured parasite line samples would also work with dried blood samples collected in the field.
The number of field samples originally analyzed was limited by their availability to us and by the work restrictions imposed by the COVID-19 pandemic. We increased the number of malaria field samples from Mali to n=20, and confirmed that they are correctly genotyped using the SNP assays.

Data exclusions

No data were excluded.

Replication

The replicability of HRM results was confirmed by at least 3 technical replicates per sample and at least 2 independent biological replicates.

Randomization

Not applicable.

Blinding

Blinding was not practical in the experimental design. The person running the PCR was working by herself and had to identify the samples before qPCR runs.

Reporting for specific materials, systems and methods

We require information from authors about some types of materials, experimental systems and methods used in many studies. Here, indicate whether each material, system or method listed is relevant to your study. If you are not sure if a list item applies to your research, read the appropriate section before selecting a response.

Materials & experimental systems

n/a	Involved in the study	
☒	☐	Antibodies
☒	☐	Eukaryotic cell lines
☒	☐	Palaeontology and archaeology
☒	☐	Animals and other organisms
☐	☒	Human research participants
☒	☐	Clinical data
☒	☐	Dual use research of concern

Methods

n/a	Involved in the study	
☒	☐	ChIP-seq
☒	☐	Flow cytometry
☒	☐	MRI-based neuroimaging

Human research participants

Policy information about studies involving human research participants

Population characteristics

The only characteristic relevant for our study was that the participants were infected with malaria in Africa, at the time of sample collection.

Recruitment

The individual samples were selected randomly from a large cohort.

Ethics oversight

The ethics committee of the Faculty of Medicine, Pharmacy and Dentistry at the University of Sciences, Techniques and Technology of Bamako, and the Institutional Review Board of NIAID, NIH approved the Mali study (NIH protocol number 11-I-N126; ClinicalTrials.gov NCT01322581). Written, informed consent was obtained from the parents or guardians of participating children or from adult participants.

Note that full information on the approval of the study protocol must also be provided in the manuscript.
